# Validity of self-reported versus actual age in Nepali children and young people

**DOI:** 10.1016/j.puhe.2016.02.014

**Published:** 2016-08

**Authors:** M. Heys, T. Candler, A. Costello, D.S. Manandhar, R.M. Viner

**Affiliations:** a Institute for Global Health, University College London, UK; b Department of Paediatrics Bristol Royal Infirmary, University Hospitals Bristol NHS Trust, UK; c Department of Maternal, Newborn, Child and Adolescent Health (MCA), World Health Organization, Switzerland; d Mother and Infant Research Activities (MIRA), Nepal; e Institute of Child Health, University College London, UK

## Abstract

• Self-reported age is a potential source of misclassification bias in International Surveys.
• We compare objectively recorded age with self-reported age at mean age 11.5 years in 3943 children in rural Nepal.
• There was high agreement between actual and self-reported age with an error rate of 7%.

Many international surveys of children and young people rely on age reported by parents or by young people.^1^ Recording of births is not standardised, age may be reported approximately, and in cultures such as Nepal, South Asia, age may be reported in ‘running’ years, such that a child age 10 years will say they are ‘running 11’. So self-reported age is potentially an important source of misclassification bias. There is a significant body of evidence examining the potential for misclassification bias in the report of health measures and health related behaviours, such as weight,^2^ age of menopause^3^, ^4^ and smoking.^5^ However, the accuracy of reported versus actual age has not been tested – there is no published study that compares actual with self-reported age in low income country settings.

The aim of this study was to assess the validity of self-reported age in a rural low income setting. Using data from a closed adolescent birth cohort study we compared objectively recorded age with self-reported age in full years in a group of 3943 children aged between recorded ages of 9.5 and 13.1 years (mean 11.5 years).

The mothers of the children and young people included in this cohort were part of a cluster randomized control trial of a community based intervention – women's participatory groups – in rural Makwanpur, Nepal.^6^ All women who were pregnant during the trial period, Oct 2001 to Nov 2003, were invited to take part in the women's groups and around one third of this population chose to do so. The women's groups addressed issues around pregnancy, childbirth, and newborn and child health. The main outcomes, neonatal and maternal mortality, were assessed at four weeks postpartum by household interview for all women (both those who attended and those who did not attend women's groups). Maternal and neonatal mortality were significantly lower in intervention than control clusters. Date of birth was collected from the families by a field interviewer within four weeks of birth.

An average of 11.5 years later the first and only long term follow-up of this cohort was conducted (2014–2015). Surviving children who consented and were traceable at the time of follow-up underwent a face-to-face interview in their homes. During the interview children were asked to give their self-reported age. A family member, usually their mother, was present at all times during the interview. Self-reported age was recorded in full years.

The perinatal and long term follow-up data from this cohort were matched to create a closed adolescent birth cohort. Retention rate for surviving infants at four weeks of age to mean 11.5 years was 73%. An additional 3.2% of the original cohort died between trial completion and long term follow-up and 4.6% of the original cohort had missing age data (Fig. 1).

From the date of interview and the date of birth we calculated ‘true’ age in years – termed objectively recorded age. These were then categorized into complete years for example 10 years of age included all those with ages between 9.999 years and 10.999 years. Chi-squared and kappa tests were used to compare reported age groups with true age groups. Bland Altman plots were also used to assess measurement agreement.

The table shows around 90–95% agreement with significant correlation between actual and self-reported age (*P* < 0.001 for Chi^2^, Kappa value 0.89). Overall agreement between self-reported and recorded age was 93.1%. The overall error rate was therefore 6.9% (95% confidence interval, 6.1 to 7.7). The limits of agreement between each measure of age were −0.52–0.57 years. Self-reported age was greater than actual age by a mean difference of 0.02 years (95% CI 0.01 to 0.03). Pitman's Test of difference in variance was r = 0.014 (*P* = 0.394) Pitman's test examines for the equality of variance between two correlated samples, with the null hypothesis rejected here being that there is a significant difference in the variances of the two samples (Table 1).

Strengths of our study are that we have reliable birth dates and interview dates in a resource poor setting. Limitations are that interviews were carried out with family members present and therefore we cannot be certain whether reported age was that from the child alone or from the family member present at interview. This would limit the generalizability of our findings to surveys that are carried out with interviews taking place solely with the child or young person.

There was high agreement between reported and actual age. While it is reasonable to rely on reported age for national and international epidemiological surveys in low income settings like Nepal, our study did show an error rate of 6.9% (95% CI 6.1 to 7.7) which might influence overall estimates of nutritional status, immunization coverage, puberty scores and other child health variables.

## Author statements

### Acknowledgements

First and foremost we thank the families, mothers and children and young people of the Makwanpur women's group study who gave their time generously and without complaint both during the initial trial and the subsequent follow-up study. Additionally we thank the field staff of the MIRA Makwanpur study team, in particular Kirti Man Tumbahangphe, Dej Krishna Shrestha, Dhruba Adhikari, Bharat Budathoki, Sagar Khadka and Aman Sen. We thank Professor Ramesh Kant Adhikari and Dr. Naomi Saville for their guidance on the follow-up study and Dr. Melissa Neuman for her data cleaning of the original trial dataset.

### Ethical approval

Ethical approval for the original trial was obtained from the Nepal Health Research Council and the ethics committee of the Institute of Child Health and Great Ormond Street Hospital for Children. Ethical approval for the follow-up study was obtained from the Nepal Health Research Council (Reg.no.L99/2OL3) and the University College London Research Ethics Committee (Project ID: 5143/002).

### Funding

MH is funded as an Academic Clinical Lecturer by the National Institute for Health Research, UK. The follow-up study field work was funded as part of a Wellcome Trust Strategic Award (WT085417MA).

### Competing interests

None to declare.

### Author contributions

MH, RV conceived the idea for this paper. MH conducted the follow-up field study in Nepal under supervision of AC and DSM. AC and DSM first conceived the idea of the follow-up study and also conducted the initial cluster Randomised Control Trial with baseline data as used in this paper. TC reviewed and edited the manuscript. MH conducted the analyses. MH wrote first draft of the paper. MH, AC, DSM, TC and RV all contributed to interpretation of the analysis, edited the final manuscript and approved the manuscript in the final format.

## Figures and Tables

**Fig. 1:**
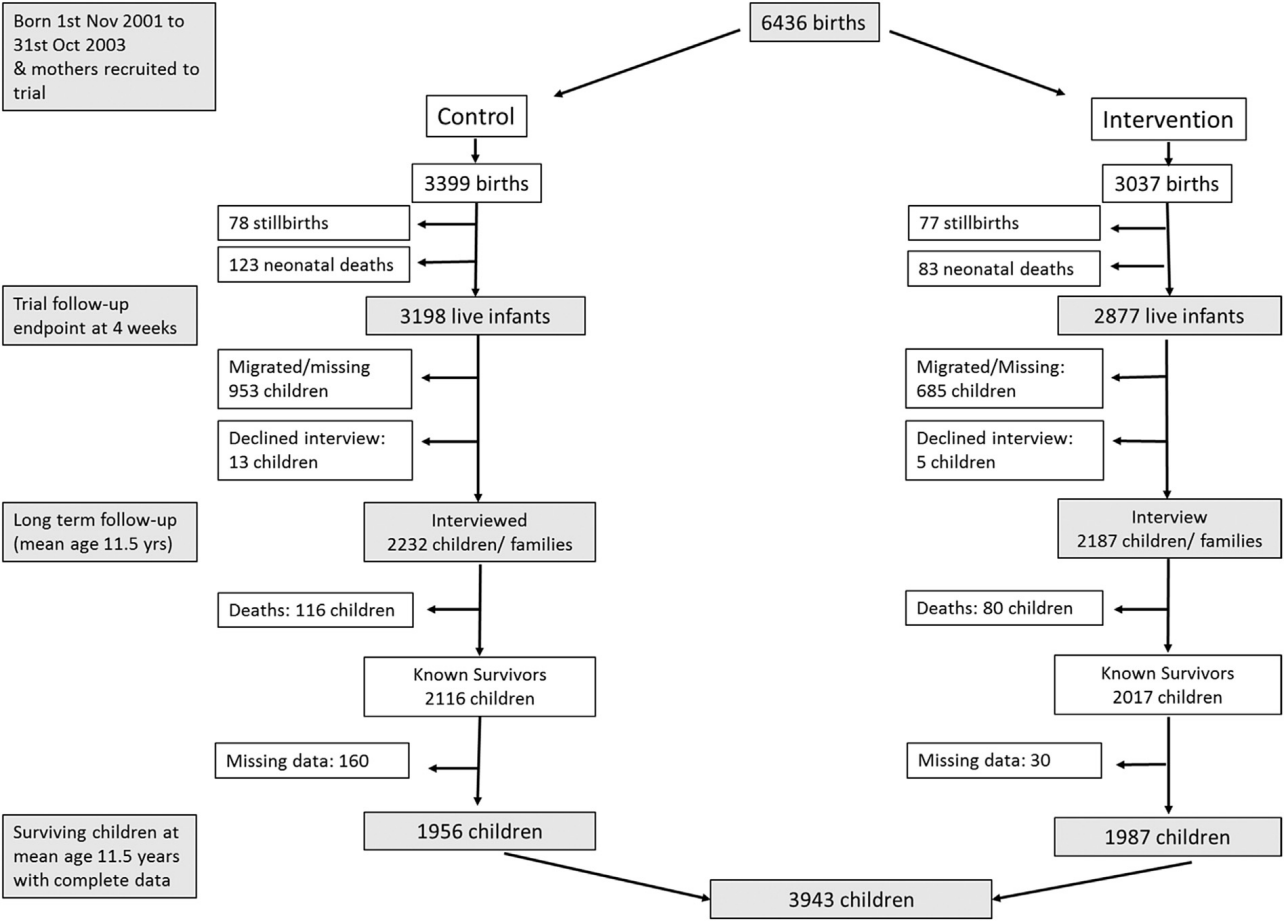
Trial participant flow chart, follow-up mean 11.5 years later and number of children with data on both self-reported and acutal age.

**Table 1:** Percentage agreement and numbers of children and young people between reported and actual age (in years) for 3943 girls and boys in rural Nepal.

		Self-reported age (full years)			
9	10	11	12
Recorded age (full years)	9	0% (*n* = 0)	0.1% (*n* = 1)	0% (*n* = 0)	0% (*n* = 0)
10	100% (*n* = 5)	95.2% (*n* = 993)	4.3% (*n* = 88)	0.8% (*n* = 7)
11	0 (*n* = 0)	4.7% (*n* = 49)	93.7% (*n* = 1914)	9.4% (*n* = 80)
12	0% (*n* = 0)	0% (*n* = 0)	2.0% (*n* = 41)	89.7% (*n* = 764)
13	0% (*n* = 0)	0% (*n* = 0)	0% (*n* = 0)	0.1% (*n* = 1)
**Overall figure of agreement**		**93.1%**			
